# Detection of knockdown resistance (*kdr*) mutations in *Anopheles gambiae*: a comparison of two new high-throughput assays with existing methods

**DOI:** 10.1186/1475-2875-6-111

**Published:** 2007-08-13

**Authors:** Chris Bass, Dimitra Nikou, Martin J Donnelly, Martin S Williamson, Hilary Ranson, Amanda Ball, John Vontas, Linda M Field

**Affiliations:** 1 Department of Biological Chemistry, Rothamsted Research, Harpenden, AL5 2JQ, UK; 2 Vector Group, Liverpool School of Tropical Medicine, Pembroke Place, Liverpool L35QA, UK; 3 Division of Cell and Molecular Biology, Sir Alexander Flemming Building, Imperial College, London, UK; 4 Laboratory of Pesticide Science, Agricultural University of Athens, Iera Odos 75, 118 55, Votanikos, Athens, Greece

## Abstract

**Background:**

Knockdown resistance (*kdr*) is a well-characterized mechanism of resistance to pyrethroid insecticides in many insect species and is caused by point mutations of the pyrethroid target site the *para*-type sodium channel. The presence of *kdr *mutations in *Anopheles gambiae*, the most important malaria vector in Africa, has been monitored using a variety of molecular techniques. However, there are few reports comparing the performance of these different assays. In this study, two new high-throughput assays were developed and compared with four established techniques.

**Methods:**

Fluorescence-based assays based on 1) TaqMan probes and 2) high resolution melt (HRM) analysis were developed to detect *kdr *alleles in *An. gambiae*. Four previously reported techniques for *kdr *detection, Allele Specific Polymerase Chain Reaction (AS-PCR), Heated Oligonucleotide Ligation Assay (HOLA), Sequence Specific Oligonucleotide Probe – Enzyme-Linked ImmunoSorbent Assay (SSOP-ELISA) and PCR-Dot Blot were also optimized. The sensitivity and specificity of all six assays was then compared in a blind genotyping trial of 96 single insect samples that included a variety of *kdr *genotypes and African *Anopheline *species. The relative merits of each assay was assessed based on the performance in the genotyping trial, the length/difficulty of each protocol, cost (both capital outlay and consumable cost), and safety (requirement for hazardous chemicals).

**Results:**

The real-time TaqMan assay was both the most sensitive (with the lowest number of failed reactions) and the most specific (with the lowest number of incorrect scores). Adapting the TaqMan assay to use a PCR machine and endpoint measurement with a fluorimeter showed a slight reduction in sensitivity and specificity. HRM initially gave promising results but was more sensitive to both DNA quality and quantity and consequently showed a higher rate of failure and incorrect scores. The sensitivity and specificity of AS-PCR, SSOP-ELISA, PCR Dot Blot and HOLA was fairly similar with a small number of failures and incorrect scores.

**Conclusion:**

The results of blind genotyping trials of each assay indicate that where maximum sensitivity and specificity are required the TaqMan real-time assay is the preferred method. However, the cost of this assay, particularly in terms of initial capital outlay, is higher than that of some of the other methods. TaqMan assays using a PCR machine and fluorimeter are nearly as sensitive as real-time assays and provide a cost saving in capital expenditure. If price is a primary factor in assay choice then the AS-PCR, SSOP-ELISA, and HOLA are all reasonable alternatives with the SSOP-ELISA approach having the highest throughput.

## Background

Members of the *Anopheles gambiae *complex are the major vectors of malaria in sub-Saharan Africa. One of the most effective vector-directed malaria control strategies involves the use of insecticide-treated bednets (ITNs) [1-4]. The only class of insecticides presently licensed for this purpose are the pyrethroids which show low mammalian toxicity and fast knockdown activity. Unfortunately, the intensive use of pyrethroids, including their indirect use in agriculture, has led to reports of reduced efficacy [5,6].

Pyrethroids act on the insect nervous system by altering the normal function of the *para*-type sodium channel, resulting in prolonged channel opening that causes increased nerve impulse transmission, leading to paralysis and death [7,8]. Resistance to pyrethroids is often associated with alterations (point mutations) in the *para*-type sodium channel gene, that cause reduced neuronal sensitivity. This resistance mechanism was first identified in the house fly *Musca domestica *and was termed knockdown resistance or *kdr *[9]. Subsequent analyses demonstrated that *kdr *was caused by a leucine to phenylalanine (L1014F) replacement in transmembrane segment 6 of domain II of the sodium channel [10]. Two amino acid substitutions at the same position (L1014F and L1014S) have been reported in pyrethroid resistant *An. gambiae *initially in *An. gambiae s.s. *[11,12] and more recently in *Anopheles arabiensis *[13,14]. In several West African countries the predominant *kdr *mutation in *An. gambiae *populations is the leucine to phenyalanine substitution (L1014F) termed *kdr *west (*kdr-*w), whilst in East African populations the leucine to serine (L1014S) termed *kdr *east (*kdr*-e) is more common [14-19]. Recently, individuals heterozygous for both the *kdr*-w and *kdr*-e alleles have been reported [20,21].

Sensitive detection of the mutations associated with resistance is a prerequisite for resistance management strategies aimed at prolonging insecticide life while maintaining sufficient insect control. This type of monitoring requires rapid high-throughput assays and there are currently several different methods available for detecting the DNA changes responsible for *kdr *in *An. gambiae*. The most widely used method is based on Allele Specific PCR (AS-PCR) [11,12], but more recently a number of other assays have been described including Heated Oligonucleotide Ligation Assay (HOLA) [22], Sequence Specific Oligonucleotide Probe Enzyme-Linked ImmunoSorbent Assay (SSOP-ELISA) [23], PCR-Dot Blot [24], Fluorescence Resonance Energy Transfer (FRET)/Melt Curve analysis [20] and PCR elongation with fluorescence [25]. However, to date there are few reports comparing the performance and relative advantages and disadvantages (safety, cost, speed, simplicity etc.) of these assays under comparable conditions. Here a single blind comparison of the performance of four of these assays with two newly developed fluorescence-based high-throughput assays (TaqMan and High Resolution Melt – HRM) was carried out using a 96 sample reference plate containing DNAs from a variety of field-collected *Anopheles *individuals representing all the known *kdr *genotypes.

## Methods

### Mosquito collections and preparation of 96 sample reference plate

For the initial optimisation of each assay mosquitoes were either obtained from two laboratory colonies, Kisumu (susceptible line from Kenya) and RSP (homozygous for the East African *kdr *mutation), or were field-caught samples from Burkina Faso, Ghana, Kenya and Cameroon. Genotypes of individuals were confirmed by sequencing of the relevant region of the *para*-type sodium channel gene as described previously [12].

All detection assays were performed on a standard 96 well test plate. The 96 sample test plate was comprised of genomic DNA of representative mosquito individuals of all the known *kdr *genotypes including three individuals heterozygous for both the east and west *kdr *alleles. The plate included DNA from *An. gambiae s.s *(both S and M forms) *An. arabiensis, Anopheles quadriannulatus, Anopheles melas, Anopheles merus *and *Anopheles funestus*. The amount of DNA was variable between samples to test the sensitivity of each assay. DNA concentration was determined by absorption at 260 nm using a NanoDrop spectrophotometer (NanoDrop Technologies). The plate also included a number of *Plasmodium falciparum *DNA samples and water blanks as negative controls. The details of each of the 96 samples (including species, molecular form, collection location, DNA concentration and *kdr *genotype) is given in Additional file 1. This information was withheld from the persons who carried out the testing of each assay to ensure no bias occurred in the scoring of results. For all samples DNA was extracted from single mosquitoes using either the Livak or Ballinger Crabtree methods [26,27] or DNAzol reagent (Molecular Research Center, Inc) at one-fifth the recommended reagent volume for each extraction. The DNAs were resuspended in either TE buffer or sterile water at volumes between 100 and 200 μl. Species identification was carried out using an established PCR assay [28] and specimens had been assigned a putative *kdr *genotype by AS-PCR [11,12], HOLA [22] or DNA sequencing. After the blind genotyping trials any samples of ambiguous *kdr *genotype were sequenced.

### AS-PCR

AS-PCR was carried out following the methods in the original descriptions of AS-PCR for *kdr *detection in *Anopheles *[11,12]. Two reported methods of modifications to these assays were also investigated [20,29]. All four protocols were performed using three different DNA polymerases/master mixes, Dynazyme II (Finzymes), PCR master mix (Promega) HotStarTaq Plus Master Mix Kit (Qiagen) and three different PCR machines a GeneAmp™ PCR System 2700 a Techne Genius and a Techne Progene. In all cases amplifications were performed in 25 μl reactions using 1 μl template. After comparison of all protocols/polymerase kits the protocol of Verhaeghen *et al *[20] using the 2 × PCR master mix (Promega) was selected to genotype the 96 sample reference plate.

### TaqMan

Previous work characterizing the *para *gene region encoding domain II S4–S6 of the sodium channel from a range of insect species has shown that this region contains an intron very close to the *kdr *mutation site. In many insect species this intron shows a degree of variation (nucleotide substitutions or insertions/deletions) between different stains/isolates which would affect the performance of any assay that uses primer binding sites within this region. Therefore, nucleotide alignments of all the *An. gambiae *and *An. arabiensis *domain II sodium channel gene sequences available in the National Center for Biotechnology Information (NCBI) database were compared and a region around the *kdr *site which was conserved in all isolates/species was selected for primer/probe design.

Forward and reverse primers and three minor groove binding (MGB) probes (Applied Biosystems) were designed using the Primer Express™ Software Version 2.0. Primers *kdr*-Forward (5'-CATTTTTCTTGGCCACTGTAGTGAT-3'), and *kdr*-Reverse (5'-CGATCTTGGTCCATGTTAATTTGCA-3') were standard oligonucleotides with no modification. The probe WT (5'-CTTACGACTAAATTTC-3') was labelled with VIC at the 5' end for the detection of the wildtype allele, the probes *kdr*W (5'-ACGACAAAATTTC-3') and *kdr*E (5'-ACGACTGAATTTC-3') were labelled with 6-FAM for detection of the *kdr*-w and *kdr*-e alleles respectively. Each probe also carried a 3' non-fluorescent quencher and a minor groove binder at the 3' end. The minor groove binder provides more accurate allelic discrimination by increasing the T_M _between matched and mis-matched probes [30]. The primers *kdr*-Forward and *kdr*-Reverse and the WT probe were used in one assay with probe *kdr*W for *kdr*-w detection and in a second assay with probe *kdr*E for *kdr*-e detection.

PCR reactions (25 μl) contained 1 μl of genomic DNA, 12.5 μl of SensiMix DNA kit (Quantace), 900 nM of each primer and 200 nM of each probe. Samples were run on a Rotor-Gene 6000™ (Corbett Research) using the temperature cycling conditions of: 10 minutes at 95°C followed by 40 cycles of 95°C for 10 seconds and 60°C for 45 seconds. The increase in VIC and FAM fluorescence was monitored in real time by acquiring each cycle on the yellow (530 nm excitation and 555 nm emission) and green channel (470 nm excitation and 510 emission) of the Rotor-Gene respectively.

The TaqMan assays were also performed using a standard PCR machine followed by endpoint measurements using a fluorimeter. For this the PCR reactions were set up as described above and run on a GeneAmp™ PCR System 2700 (Applied Biosystems) using temperature cycling conditions of: 10 minutes at 95°C followed by 40 cycles of 92°C for 15 seconds and 60°C for 1 minute. Reactions were then transferred to black half-area microtitre plates (Costar) and read in a FLx800 fluorimeter (Biotek) using 485/20 excitation and 528/20 emission filters for FAM detection and 530/25 excitation and 560/10 emission filters for VIC detection. The sensitivity of the FLx800 was adjusted for FAM and VIC fluorescence to achieve the maximum dynamic range without exceeding the maximum threshold. To determine the background level of fluorescence of the assay three or more no template controls were included in each run and the fluorescence values of these reactions averaged and subtracted from all values. To aid in genotype scoring a cut-off threshold was established by subtracting a further percentage of the averaged negative control value to create only positive or negative values. Percentages varied for the different probe fluorophore measurements. For the east and west assay susceptible probes labelled with VIC, an additional 15% was subtracted. For the *kdr*-w allele specific probe (VIC), an additional 20% was subtracted and for the *kdr*-e specific probe, an additional 60% was subtracted. The cut-off values given here were found to work well with the master mix and conditions described above. However, when using different master mixes or fluorimeters the cut-off thresholds were found to vary, so if alternative conditions are to be used optimization with templates of known genotype may be required.

### HRM

The design of a HRM assay for *kdr *detection followed the recommendations in previous reports of this technique [31-33]. The same forward and reverse primers (*kdr*-Forward and *kdr*-Reverse) that were used in the TaqMan assay were also used for HRM as they efficiently amplified a small product of 71 bp. PCR reactions contained 1 μl of genomic DNA, 12.5 μl of SensiMix DNA kit (Quantace), 300 nM of each primer and 1.5 μM of SYTO 9 (Invitrogen) made up to 25 μl with filter sterilized water. Samples were run on a Rotor-Gene 6000 (HRM)™ (Corbett Research) using temperature cycling conditions of: 10 minutes at 95°C followed by 40 cycles of 95°C for 5 seconds and 60°C for 10 seconds. This was followed by a melt step of 65–75°C in 0.1°C increments pausing for 2 seconds per step. The increase in SYTO 9 fluorescence was monitored in real time during the PCR and the subsequent decrease during the melt phase by acquiring each cycle/step to the green channel (470 nm excitation and 510 nm emission) of the Rotor-Gene. Genotypes were scored by examining normalized and difference melt plots using the Rotor-Gene Software.

### HOLA

HOLA was carried out following the protocol of Lynd *et al *[22] with no modification.

### SSOP-ELISA

SSOP-ELISA followed the protocol of Kulkarni *et al *[23] with slight modifications. Primers AgD1 and AgD2 primers [23] were used to PCR amplify a 293 bp fragment from domain II of the sodium channel gene. The primer AgD2 carries a biotin modification at the 5' end. PCR was carried out in a 25 μl volume with a final concentration of 1 × Buffer, 2 mM MgCl_2_, 0.2 mM dNTPs, 0.1 μM each primer, 0.034 U/μl Taq DNA polymerase (Qiagen). Reaction conditions were 94°C for 5 min followed by 35 cycles of 94°C for 30 sec, 48°C for 40 sec, 72°C for 40 sec and a final extension step of 72°C for 10 min. PCR products were diluted 1:1 in water, denatured at 95°C for five minutes and cooled to 4°C. The 3' end digoxigenin-conjugated SSOPs (104F, 104S, 104L) [22] were added together with the diluted PCR products to the streptavidin-coated ELISA plates (Sigma) as described previously [22]. Washes were performed [22] and 100 μl of TMB substrate (Roche, 11 484 281 001) was added. After five minutes, the reaction was stopped with the addition of 0.5 M H_2_SO_4 _and the optical density at 450 nm was measured in an ELISA reader.

### PCR-Dot Blot

PCR was carried out as for the SSOP-ELISA method (although in this case the AgD2 primer is unmodified). Amplified DNA products were denatured for 2 min at 94°C and then cooled to 4°C. One and a half μl of PCR products were spotted onto nylon membranes (Roche) and fixed to them by cross-linking with ultraviolet radiation. Membranes were probed with the 104L, 104F and 104S probes [22] at 42°C for 1.5 hours and then washed twice with 2 × SSC 0.1% SDS for 5 min at room temperature, followed by two washings with 0.2 × SSC 0.1% SDS at 42°C for the three *kdr *alleles (15 minutes per wash). Membranes were then placed in blocking buffer (Roche) for 30 minutes. Probes were detected using a non-radioactive CSPD substrate (Roche) based approach. Alkaline phosphatase-conjugated anti-digoxigenin Fab fragments and CSPD substrate were added to the membranes following manufacturer's instructions. Membranes were finally exposed to Hyperfilm ECL for approximately 6 hours.

## Results

### AS-PCR

In allele-specific PCR oligonucleotide primers that are designed to be allele specific (by incorporating sequence specific differences between alleles into the 3' end of the primers) are used in PCR in combination with allele non-specific primers to amplify allele-specific and allele non-specific 'control' fragments of different sizes. Agarose gel electrophoresis is carried out post-PCR to separate the DNA fragments and score genotypes [34].

**Figure 1:**
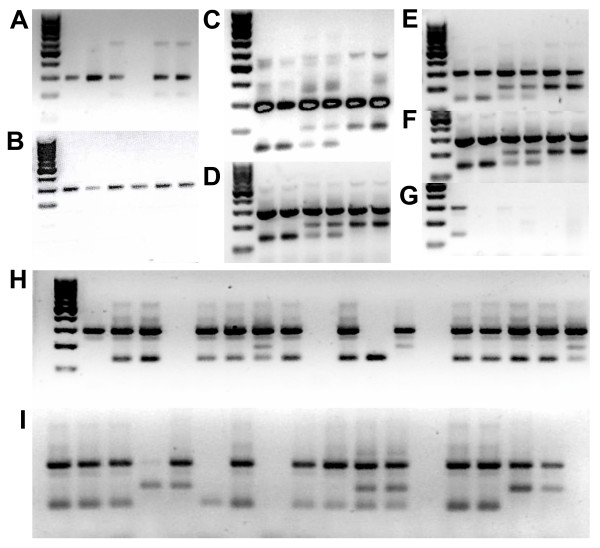
**Examples of AS-PCR products for *kdr*-e and *kdr*-w genotyping**. Gels A to D show examples of AS-PCR results using four different protocols, A [29], B [11], C [12], D [20]. Gels E to G show the result of using different DNA polymerases on the AS-PCR method described by [20], E: Promega PCR master mix, F: Qiagen HotStar Taq G:Finzymes Dynazyme II Gel H is an example of results of using the protocol of [20] with the Promega PCR master mix to genotype samples using the *kdr*-e assay from the 96 reference plate and gel I for the *kdr*-w assay. The same DNA templates were used in PCRs shown in gels A to G and from left to right were 100 bp DNA Ladder (Fermentas), homozygous wildtype, homozygous wildtype, heterozygous, heterozygous, homozygous mutant, homozygous mutant.

A number of different AS-PCR protocols were investigated using several enzyme mixes giving variable results (Figure 1). The best results were achieved following the protocol of Verhaeghen *et al *[20]. This method differs from the others in that it uses a convenient combined temperature cycling program for both east and west *kdr *assays. Both the Promega PCR and Qiagen HotStar master mixes gave good results (Figure 1), although the Qiagen mix gave slightly greater amplicon yield the Promega mix is cheaper and was therefore chosen to genotype the 96 sample reference plate. Banding patterns were scored by eye and the results are shown in Table 1 and Additional file 1. The results indicate this method is reasonably sensitive with 15 failed reactions (where a genotype could not be determined). Of these four were DNA from *An. funestus *(which is either very degraded or contains strong inhibitors, see the TaqMan results section) and four from *An. merus *individuals. The AS-PCR method was also reasonably accurate with only three incorrect scores, probably resulting from difficulties in visual scoring which was affected by variation in the quality of the agarose gels.

**Table 1:** Performance of seven assays in the blind *kdr *genotyping trial. Table shows the number of samples scored correctly, failed to score, or incorrectly scored out of a total of 96 samples.

	***TaqMan***	***TaqMan Endpoint***	***AS-PCR***	***SSOP-ELISA***	***PCR Dot Blot***	***HOLA***	***HRM***
**Correct scores**	91	87	78	78	79	77	73
**Failed reactions**	5	7	15	13	12	8	16
**Misscores**	0	2	3	5	5	11	7

### TaqMan

The TaqMan assay is a PCR method employing oligonucleotide probes that are dual-labelled with a fluorescent reporter dye and a quencher molecule. Amplification of the probe-specific product causes cleavage of the probe, generating an increase in reporter fluorescence as the reporter dye is released away from the quencher. By using different reporter dyes, cleavage of allele-specific probes can be detected in a single PCR [35].

**Figure 2:**
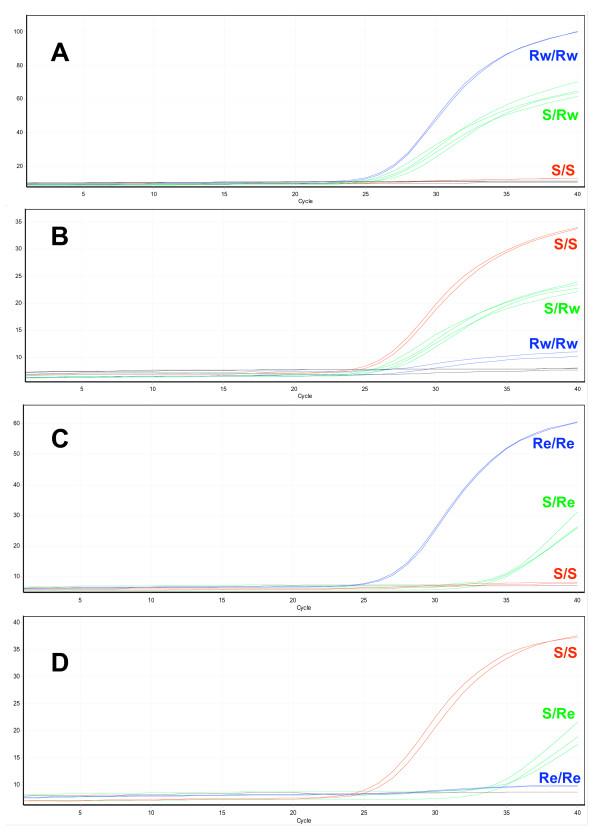
**Real-time TaqMan detection of the *kdr*-e and *kdr*-w alleles**. A) and B) Detection of the *kdr*-w mutation. C) and D) Detection of the *kdr*-e mutation. A) Cycling of FAM-labelled probe specific for the *kdr*-w allele. C) Cycling of the FAM-labelled probe specific for the *kdr*-e allele. B) and D) cycling of the VIC labelled probe specific for the wild type allele. S: Wild type allele (L1014), Rw: Resistant allele, West African mutation (L1014F), Re: Resistant allele, East African mutation (L1014S).

**Figure 3:**
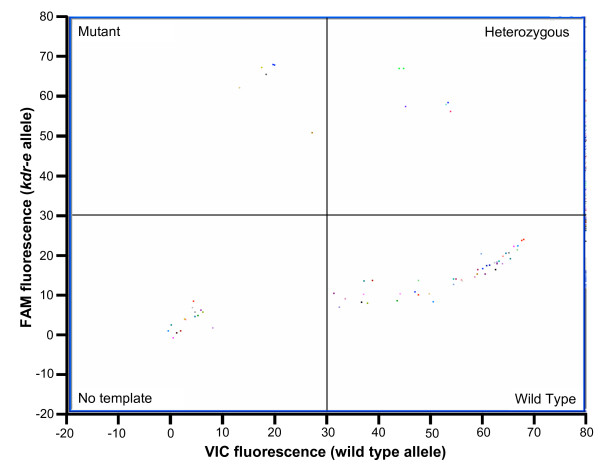
**Scatter plot analysis of TaqMan fluorescence data**. In this example real time PCR was carried out using the east *kdr *assay on ~70 samples from the 96 samples reference plate then fluorescence values of the FAM labelled probe specific for the *kdr*-e mutation were plotted against the VIC labelled probe specific for the wild type allele.

After minimal optimization using templates of known genotype both the *kdr*-e and *kdr*-w TaqMan assays showed excellent discrimination of the two resistance alleles. Both assays use two probes, the first specific for the wildtype allele is labelled with VIC and the second, specific for the mutant allele (*kdr*-w or *kdr*-e), is labelled with FAM. In either assay a substantial increase in VIC fluorescence indicates a homozygous wildtype, a substantial increase in FAM fluorescence indicates a homozygous mutant and a, usually intermediate, increase in both signals indicates a heterozygote (Figure 2). Individuals homozygous for the *kdr*-e mutation display no increase in VIC or FAM fluorescence in the *kdr*-w assay and vice versa. To help score the genotypes the Rotor-Gene software allows endpoint fluorescence values for the two dyes to be automatically corrected for background and plotted against each other in bi-directional scatter plots (Figure 3). The clustering of samples in scatter plots in addition to the real-time fluorescence traces gives easy and accurate genotype scoring. The results of genotyping the 96 samples in the reference plate are given in Table 1/Additional file 1 showing that the real-time TaqMan assay is sensitive, with only five failed reactions. Of these all but one were DNA samples from *An. funestus *individuals. Attempts were made to sequence the four *An. funestus *samples on the plate but repeated attempts to amplify the region of interest were unsuccessful despite over 65 cycles (in two rounds) of PCR. These four samples came from a single source in South Africa and failed to score in any of the genotyping assays so it is likely this DNA was badly degraded or contained strong inhibitors of PCR. Although there are currently no reports of *kdr *mutations in this species *An. funestus *samples were obtained from an alternative source, sequenced and run through the TaqMan assays. In this instance, the samples were correctly scored as wildtype indicating that the TaqMan assay can be used to screen this species for the potential emergence of *kdr*. Overall the TaqMan assay demonstrated high specificity in the genotyping trial with no incorrect scores being recorded.

**Figure 4:**
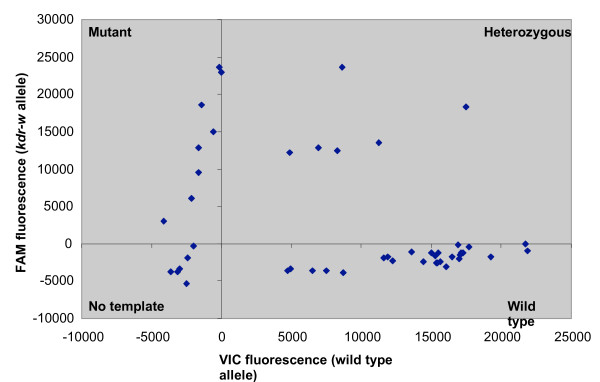
**Scatter plot analysis of TaqMan End point assay using a PCR machine+fluorimeter**. In this example PCR was carried out using the west *kdr *assay from 48 samples of the 96 sample reference plate and the fluorescence of VIC and FAM was measured on a fluorimeter. The data was corrected for background and then plotted in a bi-directional scatter plot in Microsoft Excel. Values of X and Y axes are raw fluorescence values.

The TaqMan assay can also be adapted to use a standard thermocycler followed by endpoint measurement of VIC and FAM fluorescence with a fluorimeter. Raw fluorescence values were corrected for background and an additional cut-off value (see methods) and again plotted on simple scatter plots to aid genotyping (Figure 4). The results of genotyping the 96 samples in the reference plate showed this method is both sensitive and specific (Table 1/Additional file 1) although the degree of both is slightly reduced compared with the real-time assay (two additional failed reactions and two incorrect scores).

### HRM

In HRM analyses a small region of DNA containing the mutation of interest is amplified by PCR in the presence of a third generation fluorescent dsDNA dye. The new generation of dyes for this purpose such as SYTO 9 (Invitrogen), LC Green (Idaho Technologies) and Eva Green (Biotium Inc) are less inhibitory to PCR than traditional dyes which allow them to be used at higher concentration to achieve maximum saturation of the resulting dsDNA amplicon. A high resolution melt step is then performed, centered around the T_M _of the amplicon, using machines with high optical and thermal precision. As the dsDNA dissociates into single strands the dye is released and the fluorescence diminishes giving a melt curve profile characteristic of the sequence of the amplicon [33].

**Figure 5:**
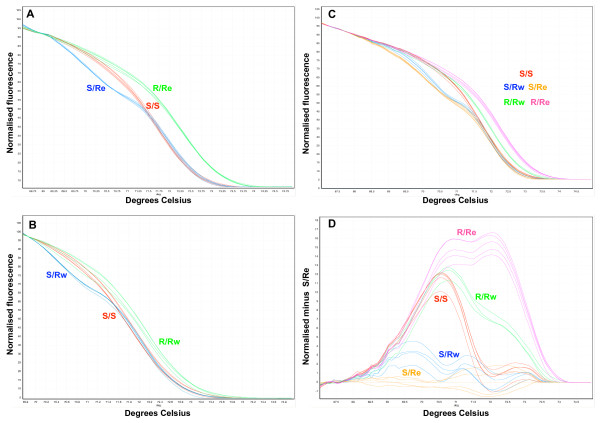
**High Resolution Melt (HRM) for detection of *kdr*-e and *kdr*-w mutations**. A) HRM detection of *kdr*-e allele. B) HRM detection of *kdr*-w allele. C) HRM detection for both *kdr*-e and *kdr*-w mutations. D) The melt curve profiles shown in C plotted as a difference plot as an aid to visual interpretation. In this case the difference in fluorescence of a sample to a selected control (in this case an S/Re genotype control) is plotted at each temperature transition. S: Wild type allele (L1014), Rw: Resistant allele, West African mutation (L1014F), Re: Resistant allele, East African mutation (L1014S).

The *kdr*-e mutation (a thymine to cytosine change) is predicted to cause a relatively large change (>0.5°C) in the melt curve T_M _of the sequence immediately surrounding it. In contrast the *kdr*-w mutation (adenine to thymine) is predicted to cause a very small change in T_M _in the melt curve making it more difficult to detect. Therefore, initial attempts were focused on optimizing HRM for detection of the *kdr*-e mutation. Using samples of known genotype HRM was able to efficiently distinguish the three possible genotypes. As shown in Figure 5A homozygous individuals were characterized by a shift in the T_M _of the melt curve whereas heterozygotes by a change in the shape of the melt curve. This change in shape results from destabilized heteroduplex annealing between some of the wild type and variant strands, creating a melt curve profile that is actually a composite of homo- and heteroduplex components [31]. The *kdr*-w mutation was subsequently optimized in the same way using samples of known genotype. As shown in Figure 5B the A/T base change produced a smaller effect on the melt curve profile, nevertheless it was still possible to distinguish homozygous individuals and due to the change in melt curve shape heterozygotes. After these promising results the effect of combining the assays was examined by looking at a number of samples covering all five possible genotypes. The results (Figure 5C) showed that the three homozygous genotypes can be distinguished by the characteristic T_M _shift in the melt curves but it was more difficult to distinguish the two heterozygous genotypes as they give similar melt curve shapes. In this case it was easier to score heterozygous individuals using the difference plot function of the Rotor-Gene software which plots the difference in fluorescence of one sample against a chosen reference at each temperature increment (Figure 5D). Despite these promising results with template DNAs of known genotype the results of using HRM to genotype the 96 samples in the reference plate were disappointing (see Table 1/Additional file 1) with a high failure rate compared to other assays (16) and a higher number of samples incorrectly scored (7). The high failure resulted from many of the samples amplifying late or failing to reach a high signal plateau in the PCR phase which gives inconclusive or low resolution HRM data. The variable amplification efficiency and incorrect scoring is likely to be due to the quantity and quality of the DNA samples.

### SSOP-ELISA

The SSOP ELISA technique combines a PCR step with subsequent visualization of products using sequence-specific oligonucleotide probes in an ELISA format. Biotinylated PCR products are captured on streptavidin-coated microtiter plates and digoxigenin-labelled sequence-specific oligonucleotide probes (SSOPs) hybridized to the PCR products. A stringent washing procedure precedes the detection of the bound SSOPs using peroxidase-conjugated anti-digoxigenin antibodies. Results can be scored by eye or quantified by spectrophotometry [36].

The results of genotyping the 96 samples in the reference plate using the SSOP-ELISA method are shown in Table 1/Additional file 1. Overall there were 13 failed reactions, four of which were DNA from *An. funestus *(which is either very degraded or contains strong inhibitors as described in the TaqMan results section) and 4 from *An. merus *individuals. The remaining five failed reactions were from *An. gambiae *DNA. One of these samples also failed in all assays apart from the TaqMan endpoint assay where it was scored as a false positive, another failed in almost all assays except for the TaqMan and the third also failed in the HOLA and HRM methods. There was some between-experiment variation in the cut-off OD values of positive and negative controls which may explain the five incorrect results that might have been caused by trivial differences in the strength of probe binding and the washing force during high stringency washes. Overall, the sensitivity and specificity of this method was similar to that displayed by the AS-PCR, PCR-dot blot and HOLA assays.

### PCR-Dot Blot

In the PCR-Dot Blot technique an initial PCR step, in which the DNA region of interest is amplified, is followed with dot-blotting of the products onto nylon membranes which are then probed with allele specific digoxigenin-labelled oligonucleotides. A stringent washing procedure precedes the detection of bound oligos using alkaline phosphatase-conjugated anti-digoxigenin antibodies. Membranes are exposed to chemiluminescent sensitive films and scored visually [37].

**Figure 6:**
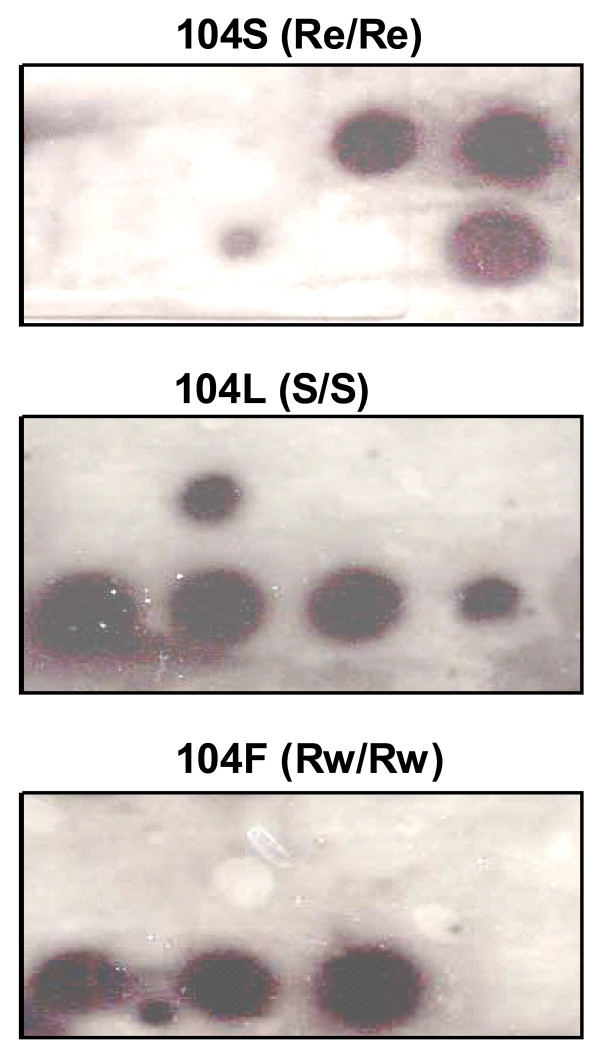
**PCR Dot blot for detection of *kdr*-e and *kdr*-w alleles**. The same reactions are shown on a portion of three membranes each probed with a different sequence specific oligonucleotide probe, 104S specific for the *kdr*-e allele, 104L specific for the wild type allele or 104F specific for the *kdr*-w allele. Reaction products shown are, top row (left to right): no template control, homozygous wild type, homozygous *kdr*-e, homozygous *kdr*-e, bottom row (left to right): heterozygous *kdr*-w/wild type, heterozygous *kdr*-w/wild type, heterozygous *kdr*-w/wild type, heterozygous *kdr*-e/wild type.

The PCR-dot blot hybridization assay has been used previously for the detection of the west *kdr *and wildtype alleles [24]. Here, a modified protocol using the DIG hybridization and detection system (Roche) for the detection of the wildtype allele and both the east and west *kdr *alleles is described. A portion of the resulting membranes showing examples of *kdr *genotyping is shown in Figure 6. The performance of this method in genotyping of the 96 samples in the reference plate is shown in Table 1/Additional file 1. The sensitivity and specificity of this method was comparable to that demonstrated by the AS-PCR and SSOP-ELISA methods. Of the 12 failed reactions, four were DNA from *An. funestus *and four from *An. merus *individuals. The remaining four reactions were DNA from *An. gambiae *individuals. From these, one reaction also failed in all assays apart from TaqMan, another failed in AS-PCR and the TaqMan endpoint assay and a third failed in all assays except for the TaqMan endpoint assay where it gave a false positive. The forth reaction failed only in the PCR dot-blot assay while an inconclusive result for this reaction was observed in the HRM assay. Incorrect scoring in five instances may be attributed to the higher background caused by differences in the strength of probe binding and the washing force during high stringency washes.

### HOLA

In the HOLA technique an initial PCR step to amplify the region of interest is followed by a hot ligation step during which ligation occurs between biotinylated "allele-specific detector" oligonucleotides and fluorescein-labelled "reporter" oligonucleotides when the 3' detector nucleotide is complementary to a nucleotide at the SNP locus. Ligated products are then captured in 96-well streptavidin plates, and successful ligation is detected using peroxidase-labelled anti-fluorescein antibodies.  Results can be scored by eye or using a 96-well microplate spectrophotometer [38].

The results of the blind genotyping of the 96 samples in the reference plate using the HOLA method are shown in Table 1/Additional file 1. The results were scored either visually or analysed on an ELISA plate reader. Overall the HOLA method genotyped a similar number of samples correctly as the AS-PCR, SSOP-ELISA and Dot Blot methods. Compared to these other assays there was a lower number of failed reactions (three of which were from *An. funestus *DNA the remainder *An. gambiae*) but a higher rate of incorrect scores (11 out of 96).

## Discussion

The development of pyrethroid resistance in *Anopheles *populations has the potential to seriously compromise malaria control efforts. A recent report examining the effectiveness of using ITNs at two sites in Benin has given clear evidence of pyrethroids failing to control an *An. gambiae *population that contains *kdr *resistance at high levels [6]. This highlights the need to monitor the spread of resistance conferring alleles and to use this information to devise management strategies to prolong the effective life of the insecticide and to help make decisions on which insecticide class to best use for effective control. There are a number of assays available for genotyping *kdr *alleles. The most widely used of these in malaria endemic countries is the AS-PCR method, probably due to its relatively low cost (both capital expenditure and running costs) (Table 2); however, a number of recent reports have questioned the reliability of this technique [20,29]. In this study, a number of protocol variations on the basic AS-PCR method were followed and the method which gave optimal results is described. The blind genotyping trial showed the AS-PCR method gave a relatively low misscore rate but compared to the TaqMan method it lacked sensitivity with a higher rate of failed reactions. The comparisons given in Table 2 also show other disadvantages of this technique; for example, the potential safety hazard presented by the use of ethidium bromide and the relatively low-throughput compared to TaqMan and HRM.

**Table 2:** Comparison of seven assays for *kdr *genotyping based on specialist equipment required, cost, safety, simplicity of protocol and speed of method. Capital cost was calculated for all assays and is correct at the time of submission. Consumable/running cost was calculated for all assays except for the HOLA and SSOP-ELISA where the running cost listed was obtained from the original report of the method.

	***TaqMan***	***TaqMan Endpoint***	***HRM***	***AS-PCR***	***SSOP-ELISA***	***PCR Dot Blot***	***HOLA***
**Specialist equipment required**	Real-time PCR machine	PCR thermocyler Fluorimeter	High-spec real time PCR machine	PCR thermocycler Gel electrophoresis and imaging equipment	PCR thermocycler Shaking incubator Multichannel pipette *optional ELISA plate reader	PCR thermocycler Shaking incubator Multichannel pipette	PCR thermocycler Multichannel pipette *optional ELISA plate reader
**Capital outlay cost **(for equipment above given in US$)	96 well $25 000 48 well $19 000	$17 800	$50 000	$10 000	$7600 *optional ELISA plate reader add $5000	$7600	$5500 *optional ELISA plate reader add $5000
**Hazardous chemicals**	-	-	-	Ethidium bromide	TMAC, H_2_SO_4_, SDS	SDS	SDS
**Protocol run time**	1 hr 45 mins	2 hrs	1 hr 35 mins	~4 hrs 30 mins	~5 hrs 30 mins	~16–18 hrs	~6 hrs 30 mins
**Number of steps**	1	2	1	2	17	16	15
**Primers/Probes required **(for detection of 3 *kdr *alleles)	2 PCR primers 3 fluorescently labelled probes	2 PCR primers 3 fluorescently labelled probes	2 PCR primers	5 PCR primers	2 PCR primers (one biotin labelled) 3 SSOPs (digoxigenin labelled)	2 PCR primers 3 SSOPs (digoxigenin labelled)	2 PCR primers 2 reporter primers (5'phosphorylation and 3'fluorescein labelled) 4 detector primers (biotin labelled)
**Number of tubes/wells/membranes required per sample**	2	2	1	2	3	3	4
**Running cost **(per sample for three alleles)	$1.72	$1.72	$0.62	$0.92	sim $1	$1.6	$1.74

Other methods with a low initial set-up cost that were investigated in this study were SSOP-ELISA, PCR DOT-BLOT and HOLA with all three giving comparable results in the blind genotyping trial. As shown in Table 2 all three assays require only basic equipment (a PCR machine, shaking incubator (ELISA) and, for the ELISA and HOLA methods, an optional ELISA reader). In addition, all dispense with the need for gel electrophoresis making them safer than AS-PCR. On the basis of cost and throughput the SSOP-ELISA method is the front-runner of the three assays. It requires approximately five and a half hours to run (17 steps) and more than 150 samples can be screened for the three *kdr *alleles in one day. The HOLA method takes approximately six and a half hours (16 steps) and 96 samples can be screened per day if four PCR machines/blocks are used. The PCR dot-blot assay can be completed in approximately 16 hours (16 steps) and more than 150 samples can be screened per day. Analysis of one sample (for the three *kdr *alleles) costs approximately US $1 using the SSOP-ELISA method, US$ 1.74 using the HOLA method and US$ 1.6 using the PCR dot-blot assay. Overall, the SSOP-ELISA, HOLA and PCR dot-blot assays require the use of basic equipment, are relatively cheap and provide acceptable sensitivity/specificity. They are thus amenable to researchers on a limited budget or without access to expensive equipment and are good options for laboratories in developing countries. The limitation of these assays lies in the requirement for a high number of post-PCR steps making them lengthier and of limited throughput capacity compared to the TaqMan and HRM assays. This is also an important consideration where operator time is included as part of the assay cost.

In this study, the performance of the four lower throughput assays was compared with two newly developed assays, TaqMan and HRM. The two high-throughput platforms both represent true closed-tube approaches requiring a single step to achieve results. This is in contrast to two recently developed high-throughput assays for *kdr *detection, FRET/MCA which requires two rounds of PCR [20] and PCR elongation with fluorescence which requires PCR followed by capillary electrophoresis [25]. HRM is a relatively new technique that has been used very successfully in a number of previous genotyping studies [32,33]. Because this method uses standard oligonucleotide primers and has no requirement for fluorescently-labelled oligonucleotides, the running costs are very low (Table 2). In addition HRM has the potential to identify novel mutations in the region of DNA encompassed by the PCR primers as any alternative base change will alter the melt profile of the amplicon. The HRM method showed initial promise during optimization with templates of known genotype (where DNA concentration was adjusted to be consistent for all samples) but subsequently performed less well in the blind genotyping trial. This is likely explained by variable DNA quality and quantity in the 96 samples in the reference plate, leading to many samples amplifying after ~35 cycles or failing to reach full plateau phase. For HRM it is recommended that the amount of DNA template used in PCR be consistent between samples as large differences in starting template will affect the observed Tm. It is, therefore, possible that this assay could be improved if DNA concentration was adjusted. However this constitutes an additional step in the protocol and would require DNA quantification using a spectrophotometer or gel electrophoresis. A comparison of HRM with the other methods (Table 2) highlights the greatest disadvantage which is the capital outlay required. Although HRM has low consumable costs it requires real-time PCR machines of high thermal and optical precision that are significantly more expensive than those that lack this specification. This high initial cost may give this assay limited application for use in resource poor malaria endemic countries.

In contrast to HRM, the TaqMan approach performed very well in the genotyping trial showing the highest level of specificity and sensitivity (as determined by the low number of failed reactions and incorrect scores) of all the assays trialled. This is likely due to both a higher degree of sensitivity and a higher tolerance to variation in DNA quality and quantity than the other assays. The TaqMan method was quick to optimise and along with HRM shows the highest throughput of the assays being simple and quick to setup (Table 2). Results can be scored easily, both manually or autoscored. The running cost of the TaqMan assay is slightly higher than the AS-PCR and HRM assays but comparable to the other methods (Table 2). Currently this method uses two separate assays to detect the *kdr*-w and *kdr*-e mutations, in future consumable costs could be further reduced by multiplexing the assay so that the wildtype, *kdr*-e and *kdr*-w alleles are detected in a single tube using probes with three fluorophores with distinct emission and excitation spectra. The other significant disadvantage of this assay is the capital outlay required, for a 96 well real-time PCR machine which costs in the region of US$ 25,000–50,000. One way to bring this cost down is through the purchase of a 48 well machine (these can be purchased for US$ 19,000–21,000) although this entails a reduction in possible throughput. An alternative cost-saving option is to carry out the TaqMan assays using a standard thermocycler for PCR and then measure endpoint fluorescence with a fluorimeter. The results described here show this is a viable approach and although a slight increase in failed reactions and incorrect scores was seen in the blind genotyping trial compared to the real-time assay, this method was still more sensitive and more specific than the AS-PCR, SSOP-ELISA, HOLA, and PCR Dot-Blots assays. A disadvantage of the endpoint method is the requirement for a small degree of data analysis before scoring (subtraction of blank and cut-off values).

Monitoring of resistance alleles such as kdr often involves the processing of thousands of individual insects per site. In this study the performance of these methods was examined on individual mosquitoes, future work could investigate the feasibility of using these assays on pooled insects to further increase throughput. A potential caveat of this approach is that it may not efficiently identify resistance alleles at low levels in mosquito populations particularly if they are present in the heterozygous state.

## Conclusion

There are a number of options available for *kdr *genotyping. In this comparison of two new high-throughput methods with four previously reported assays the TaqMan method delivered the greatest specificity and sensitivity. However, where cost is the overwhelming factor in assay choice the SSOP-ELISA method is recommended based on cost, ease of use and throughput.

## Authors' contributions

CB developed the TaqMan and HRM techniques, optimized and ran the AS-PCR method and drafted the manuscript. DN optimized and ran the SSOP-ELISA and PCR-Dot Blot methods and helped draft the manuscript. MJD helped design the study, organized and designed the reference plate of samples and helped draft the manuscript. MSW, HR and JV helped design the study and draft the manuscript. AB genotyped the reference plate using the HOLA method. LMF helped design the study and helped draft the manuscript. All authors read and approved the final manuscript.

## Supplementary Material

Additional file 1 **Excel spreadsheet showing the details of the 96 sample reference plate and full results of the blind genotyping trial**. The genotype assigned to each sample by each assay is shown. S: Wild type allele (L1014), Rw: Resistant allele, West African mutation (L1014F), Re: Resistant allele, East African mutation (L1014S). Negative controls (water or *Plasmodium falciparum *DNA) are highlighted in grey. Failed reactions are highlighted in blue. Incorrectly scored reactions are highlighted in yellow. Click here for file
